# The algorithmic artrepreneur: rethinking creative entrepreneurship under platform governance

**DOI:** 10.3389/fsoc.2026.1983042

**Published:** 2026-10-16

**Authors:** Maria Susan Mathew, Jaya Jacob, Ann Mariya Cyriac, Ajimon George, Ajo Andrews, Jerome Varghese, Jibil K. John

**Affiliations:** 1 Marian College Kuttikkanam Autonomous, Idukki, Kerala, India; 2 St. Thomas College, Kozhencherry, Kerala, India; 3 Sanathana Dharma College, Alappuzha, Kerala, India; 4 Catholicate College, Pathanamthitta, Kerala, India; 5 St. Dominic's College, Kanjirapally, Kerala, India

**Keywords:** algorithmic governance, artrepreneurship, creative labor, creative work, entrepreneurial agency, platform work, platformization

## Abstract

Digital platforms have revolutionized creativity in many ways, offering new possibilities for audiences to access, discover, engage with, and purchase artistic work. Meanwhile, the platformization of the production of creative content has introduced algorithmic systems that increasingly mediate visibility, content circulation, audience reach, and economic opportunity. This Mini Review brings together research on arts entrepreneurship, creative work, platformization, algorithmic governance and creator labor to discuss the impact of these changes on the agency of arts entrepreneurs. It highlights the possibility of platforms to lower some traditional obstacles to artistic entrepreneurship, but also to create new forms of infrastructural dependence, namely in terms of algorithmic visibility, metricisation, platform norms and monetization systems. The review suggests that contemporary artrepreneurs operate through constrained and negotiated agency, interpreting, adapting to, and sometimes challenging platform conditions. The notion of the algorithmic artrepreneur is introduced to describe an artrepreneur whose entrepreneurial agency is reconfigured through continuous negotiation with algorithmically mediated platform infrastructures affecting visibility, audience access, content circulation and value creation. This mini review conceptualizes this agency through four interrelated dimensions: creative agency, platform agency, relational agency and entrepreneurial agency. Three tensions are highlighted: the tension between visibility as an entrepreneurial opportunity and as a form of labor, the tension between platform adaptation as an entrepreneurial strategy and as a creative constraint, and the tension between digital access as a democratization and as an infrastructural dependence. There is a need to look into algorithmic agency, visibility labor, inequality, platform diversification and sustainable creative careers in the future.

## 1 Introduction

The relationship between artistic work and entrepreneurship has changed substantially as artists increasingly undertake activities associated with marketing, networking, branding, audience development, opportunity recognition, and income generation. Arts entrepreneurship scholarship has challenged the traditional separation between artistic and commercial work (Hausmann and Heinze, 2016; Patten, 2016). Arts entrepreneurship is not solely about commercializing creative output; it involves negotiating multiple forms of artistic, cultural, social, and economic value. Increasingly, this literature views entrepreneurial activity as embedded within contemporary creative careers rather than as an activity externally imposed on artistic practice.

This has been accelerated by digitalization. Social media and other digital platforms provide artists with the opportunity to connect with their audience directly, showcase their artistic works, build an online community, connect with other artists, and find new ways to monetize their work. Previous studies have focused on experimentation, promotion and access to markets with digital tools (Griffey, 2014) and more recent arts entrepreneurship scholarship on platform careers, shifting context of arts and culture work, and sustaining creative careers (Clague, 2024; Skaggs et al., 2025). Recent studies have also highlighted the importance of the cultural and social benefits of arts entrepreneurship in addition to the economic benefits (Martínez et al., 2026). Digital platforms have therefore become an increasingly important component of contemporary artistic careers.

This emerging literature has also increased attention to the role of social media in artrepreneurship. Empirical research shows that artists employ social media strategically to express their art, create their content, connect with the audience, network, collaborate, gain visibility, and succeed as artists (George and Mathew, 2025). This may indicate not only that digital platforms are no longer merely marketing tools but are becoming part of the infrastructure through which artistic entrepreneurship is organized. However, viewing platforms solely as entrepreneurial tools is increasingly insufficient. Platforms are not just a medium by which artists can interact with audiences; they enable the visibility of artists, arrange the order of content, gather behavioral data, regulate access to the audience, set up monetization plans, and impose platform-specific norms. Artistic entrepreneurship is therefore incorporated in a platformization of cultural production, in which the infrastructures through which artists operate are not fully under their control (Nieborg and Poell, 2018; Poell et al., 2021).

This evolution brings about an important empowerment–dependency tension. A strand of scholarship focuses on the democratizing power of digital platforms, their ability to decrease the barriers to access to their audiences, to self-promote and to participate in markets. A contrasting body of research highlights platform dependence, algorithmic visibility, metricisation, monitoring, and emerging forms of labor control. Recent scholarship on algorithmic management highlights tensions between flexibility and control, while emphasizing worker agency, adaptation, and resistance (Chigbu, 2026). These perspectives are not mutually exclusive in the context of artrepreneurship. Digital platforms may expand entrepreneurial opportunities while simultaneously reshaping the conditions under which those opportunities are pursued. However, this dynamic is not adequately reflected in arts entrepreneurship scholarship.

This shift also produces a conceptual separation of acting on a platform vs. being entrepreneurially constituted by the platform conditions. The former approach focuses on digital platforms as a means of promotion, networking or market access; the latter approach sees the potential impact of the platform infrastructure on the conditions under which visibility, access to audiences, circulation of content and monetisation is sought. This is crucial to distinguish because it takes digital adoption from the analysis to the reconfiguration of entrepreneurial agency.

The algorithmic artrepreneur is positioned at the intersection of, but is not identical to, several established concepts. Arts entrepreneurship or artrepreneurship concerns the integration of artistic and entrepreneurial practices and the creation of multiple forms of value (Hausmann and Heinze, 2016; Patten, 2016). Digital entrepreneurship focuses on entrepreneurial activities enabled or facilitated by digital technologies and platforms (Zaheer et al., 2019). Creator labor emphasizes the work involved in producing content, maintaining visibility and sustaining self-presentation in social media environments (Duffy, 2017), while platform work focuses on work organized through digital platforms and their infrastructures. Algorithmic management concerns the use of algorithmic systems to coordinate, monitor, evaluate or control work (Kellogg et al., 2019). The algorithmic artrepreneur differs from these constructs by focusing specifically on the reconfiguration of entrepreneurial agency when algorithmic platform conditions become consequential for artistic decision-making, audience relationships, visibility, content circulation and value creation. The concept therefore does not refer simply to an artist who uses digital platforms. It applies when platform conditions become a recurring consideration in creative presentation, audience development, networking, monetization, opportunity seeking or career planning.

This Mini Review addresses this emerging intersection by asking:

**How does platform governance reshape the entrepreneurial agency and working conditions of contemporary artrepreneurs?**

**Review approach:** This Mini Review does not follow a systematic-review protocol; rather, it adopts a focused interdisciplinary literature-synthesis approach. Relevant scholarship was identified through searches of Scopus and Web of Science, supplemented by Google Scholar and reference-list searches. The searches used combinations of the following representative terms: artrepreneurship, arts entrepreneurship, digital entrepreneurship, creative labor, creator labor, platformization, platform work, algorithmic governance, algorithmic management, visibility labor, and entrepreneurial agency. Foundational literature was considered alongside recent studies, with priority given to peer-reviewed literature addressing artistic and creative work in digital and platform contexts. Literature was selected based on its substantive relevance to platform mediation, creative entrepreneurship, algorithmic governance, visibility, audience relationships, creative autonomy, and entrepreneurial agency. The selected literature was synthesized thematically to identify conceptual intersections, tensions, and gaps across arts entrepreneurship, creative labor, platform studies, and algorithmic governance.

## 2 Conceptual framework: the algorithmic artrepreneur

The literature reviewed in this Mini Review is organized around the concept of the algorithmic artrepreneur. The concept does not simply refer to artists who use digital platforms, but to artist-entrepreneurs whose creative, platform, relational and entrepreneurial agency is exercised in and through algorithmically mediated platform environments. The framework positions platform governance as a broader infrastructural condition through which algorithmic systems shape visibility, content circulation, audience access, monetization and participation. These conditions produce processes of algorithmic negotiation that involve artrepreneurs' interpretations, adaptations, experiments, and sometimes resistance to platform environments.

The framework outlines four interrelated dimensions of artrepreneurial agency: creative agency, platform agency, relational agency and entrepreneurial agency. These dimensions are analytically distinct but mutually constitutive. They link algorithmic conditions with processes of audience development and value creation, that can lead to several types of value and, consequently, to career sustainability. The framework is therefore not proposed as a linear causal model. Instead, it is a recursive process where results lead to learning and feedback which then can influence platform strategies, audience development strategies, revenue models, and creative decisions.

The framework was developed through a synthesis of recurring concepts, mechanisms, and tensions identified in the literature on arts entrepreneurship, platformization, creative labor, creator labor, algorithmic governance, and entrepreneurial agency. The four dimensions of agency were kept separate analytically since each represents a different facet of entrepreneurial actions, and their interconnections show the reciprocal relationships found in the reviewed literature. The framework is thus not a causal model that has been tested in the field, but rather a conceptual synthesis.

The conceptual framework of the algorithmic artrepreneur is shown in Figure 1, and is the structure used for the literature synthesis that follows. The next sections elaborate on the framework by exploring the literature that underlies the key elements of the framework: arts entrepreneurship, platformization, visibility, algorithmic governance, platform adaptation and artistic autonomy.

**Figure 1:**
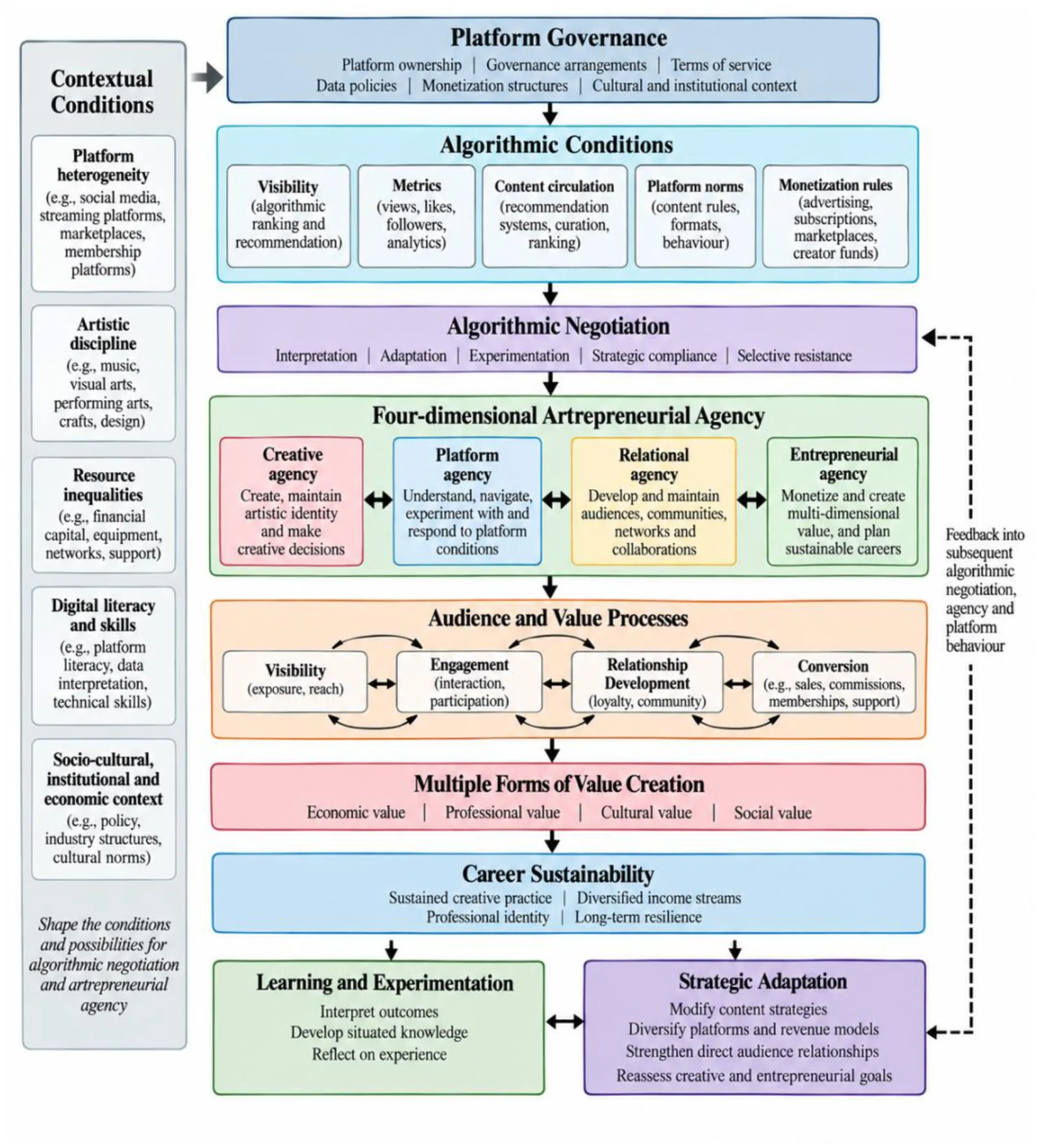
Conceptual framework of the algorithmic artrepreneur.

The framework can be interpreted as a recursive sequence, not as a linear sequence. Platform governance is influenced by contextual conditions, such as platform heterogeneity, artistic discipline, resource inequalities, digital literacy and skills, and the broader sociocultural, institutional and economic context. Platform governance sets algorithmic parameters related to visibility, metrics, content flow, platform norms, and monetization. These conditions are experienced in the algorithmic negotiation, which activates and reconfigures the four dimensions of artrepreneurial agency. Creative, platform, relational and entrepreneurial agency then engage in audience and value processes, such as visibility, engagement, relationship building and conversion. These processes can create economic, professional, cultural and social value and support career sustainability. Outcomes create learning and experimentation, which in turn leads to strategic adaptation and platform behavior.

The framework was developed by identifying patterns in the literature that were reviewed in order to identify the relationships among the recurring concepts, not by setting a causal order. There is a body of literature on arts entrepreneurship that helps to understand arts entrepreneurship and the various types of value that can be derived from an arts career. Platformization research is an approach that investigates the ways in which digital infrastructures, governance structures and economic models transform conditions of cultural production. The scholarship on visibility and creator labor points to the work of keeping content visible and building an audience, and the scholarship on algorithmic governance addresses how artists read, respond to, experiment with and engage with platform conditions. These strands were then coalesced to examine four analytically discrete but interrelated aspects of agency and the processes through which they relate to audience development, value creation and career sustainability.

## 3 From arts entrepreneurship to platformized creative work

Arts entrepreneurship developed partly because conventional entrepreneurship frameworks did not fully capture the distinctive conditions of artistic careers. Artists often pursue multiple objectives simultaneously, including creative expression, professional recognition, cultural contribution, personal fulfillment, and financial sustainability. The creative industries entrepreneur is difficult to define because the artistic and entrepreneurial identities often overlap while also reflecting distinct values (Patten, 2016). Another study shows that there is conceptual variation within the field of arts and cultural entrepreneurship (Hausmann and Heinze, 2016). Arts entrepreneurship is therefore more about a socially and institutionally situated practice of work than it is about a person's capacity to transform artistic talent into commercial products.

This complexity can be compounded by the organization of artistic careers. Often artists engage in several types of creative work at the same time, such as teaching, writing, performing, production, administration and distribution, and other ways to earn a livelihood. By building on the classic portfolio-career view, more and more artistic careers rely on a combination of creative and non-creative activities (Clague, 2024). Similarly, entrepreneurial adaptation is significant when undertaking a change in work context (Skaggs et al., 2025). The developments suggest that artistic entrepreneurship is not merely the production of artistic outputs, but requires ongoing management of constantly shifting economic, professional, and organizational circumstances as well.

Arts entrepreneurship is not just about making a profit. Community involvement, cultural identity, social cohesion, place value and entrepreneurial ecosystems have also been identified as key elements of artistic entrepreneurship (Wall-Andrews and Cukier, 2025; Martínez et al., 2026). This broader perspective situates artrepreneurship with wider social, institutional, technological and economic contexts. Digital platforms have consequently become an infrastructural component of modern artistic practice, bridging artistic production with new forms of visibility, networking and market access. This provides the broader arts-entrepreneurship context within which the more specific condition of algorithmically mediated entrepreneurship can be examined.

These viewpoints serve as the basis for two aspects of the framework: creative agency and entrepreneurial agency. Creative agency relates to the ability to keep an artistic identity and take decisions about artistic practice, and entrepreneurial agency relates to the ability to link creative and relational resources with various types of value, such as economic, professional, cultural and social value. The arts entrepreneurship literature thus sets the stage for the broader conditions of artistic and entrepreneurial action before the review examines how platform infrastructures alter the conditions of artistic and entrepreneurial action.

## 4 From social media tool to platform infrastructure

Early discussions of digital entrepreneurship frequently emphasized the empowering potential of social media. Artists would not have to deal with some of the traditional intermediaries, and could communicate directly with audiences, build their own brand, and market themselves to a geographically dispersed audience. More recent studies on artrepreneurship also show the potential benefits of social media for artistic expression and interaction with the audience, as well as networking and collaboration and commercialization (George and Mathew, 2025). The developments have increased the choices of entrepreneurial practices that artists have and have made digital engagement ever more relevant to creative careers.

But as cultural production becomes more and more platformized, this empowerment narrative becomes more complicated. Platformization therefore refers not only to the adoption of digital tools but the incorporation of platform infrastructures, governance arrangements and economic models into cultural production (Nieborg and Poell, 2018). Moreover, recent studies illustrate how platforms are increasingly structuring relationships among different market actors, such as creators, audiences, advertisers, and others (Poell et al., 2021). In the case of artrepreneurs, this implies platforms are no longer just communication spaces but are taking on a more integral role in making art visible, circulating and monetized.

This creates an important paradox. Platforms can help lessen reliance on traditional cultural intermediaries, and also can make it more likely that people rely on new, digital intermediaries. Digitalization does not eliminate gatekeeping; instead, it can make gatekeeping more data-driven and less visible. Complementary rather than competitive, algorithmic recommendation systems, platform metrics, content policies, monetization mechanisms, and audience analytics can augment traditional intermediaries like galleries, publishers, labels and agents.

Platformization thus alters the circumstances in which opportunities in art entrepreneurship are sought. The artist has more direct access to the audience but their access to infrastructures is one in which their rules, algorithms, and commercial structures are not directly under their control. The resulting relationship is therefore one of expanded access accompanied by infrastructural dependence.

This discussion corresponds to the platform-governance and algorithmic-conditions components of the framework. Platformization changes the infrastructural conditions within which artrepreneurs exercise agency by shaping visibility, audience access, content circulation, monetization, and participation. These conditions provide the context within which algorithmic negotiation and platform agency become consequential for entrepreneurial action.

## 5 The visibility paradox of artrepreneurship

Visibility is central to artistic entrepreneurship because recognition, audience interaction, commissions, sales, collaboration, and professional opportunities depend on being seen. Digital platforms can extend this visibility beyond traditional geographical and institutional boundaries. Yet maintaining visibility is becoming increasingly labor-intensive.

Creators need to consistently generate content, engage with their audience, track metrics, try out new formats and adjust strategies based on the evolving dynamics of these platforms. In the systematic review of content creation in algorithmic contexts, the following themes are highlighted: market rationality, creators' interpretations of algorithms, algorithmic control and negotiations or contestation of algorithmic power (Liang et al., 2025). For artrepreneurs, these practices go beyond the creation of art products and constitute entrepreneurial activities.

The visibility paradox is central to platformized artrepreneurship because visibility functions simultaneously as an entrepreneurial resource and as a form of labor. As a resource, visibility can generate audiences, professional opportunities, commissions and income. In terms of work, it involves a consistent production of content, self-presentation, audience management and metric monitoring.

This distinction is significant from a sociology-of-work perspective. The artists' role of promotion and relation-making becomes part of their own work when they take responsibility for their own visibility. A substantial amount of time can be spent by an artist documenting creative processes, creating content for platforms, engaging with audiences, tracking engagement, adapting future processes based on platform feedback, and so on. Digital environments can blur the boundaries between creativity, self-promotion, and work (Duffy, 2017).

Visibility should not, therefore, be equated with entrepreneurial success. It is analytically distinct from engagement, audience relationship development, monetization and sustainable career value. Exposure due to high visibility can be present without enabling lasting connections with the audience or generating economic, professional, cultural or social benefits. It is possible to make a useful distinction between visibility, relationship building, engagement, conversion and value creation. These processes are not necessarily sequential or automatic. On-platform visibility can be created without ownership and portability of audiences. Portability of the audience and less dependence on infrastructures can then be enhanced by direct channels (personal websites, mailing list, memberships, commissions, offline networks).

These processes relate especially to the relational-agency and the audience-and-value aspects of the framework. Relational agency is about building and sustaining audiences, communities, networks and collaborations. The value of being seen is thus not only a value in itself, but also a value that can start or reinforce relationships that can lead to the creation of artistic, professional, social and economic value. The difference between platform visibility and portable audience relationships also relates to the value creation and career sustainability focus of the framework, as well as to visibility work.

## 6 Algorithmic governance and entrepreneurial agency

Algorithmic systems introduce a distinctive form of uncertainty into creative work. Artists are not usually well informed about the functioning or the mechanisms of the recommendation system, nor about the reasons for the visibility of certain content. Nevertheless, entrepreneurial decisions are increasingly made within these uncertain environments.

The resulting relationship can be said to be algorithmic negotiation. Algorithmic negotiation is the ongoing process through which creators interpret, adapt to, experiment with, and sometimes resist platform systems whose operations are not fully observable or controllable. Artists can track the results of the platform, interpret metrics, try out content, tweak practice, and assess the impacts. The informal understandings of algorithmic behavior are recognized as a crucial part of contemporary content production (Liang et al., 2025).

This is similar to entrepreneurial experimentation, except that the object of experimentation is partly the platform itself. Artists try to gauge if certain types of content are being viewed, the reaction of the audience, and the potential effects of platform-specific processes on visibility. Algorithmic knowledge can then be an additional entrepreneurial competency.

Research on algorithmic management provides a broader sociological context for this development. Algorithm-based governance should, however, be differentiated from algorithmic management. In both cases, algorithmic management typically involves algorithmic systems used to organize, monitor, evaluate and/or control at work, while algorithmic governance is a broader term referring to the organization of visibility, content circulation, audience access, monetization and participation through algorithmic platforms. In this way, artrepreneurs can have algorithmic governance without being algorithmically managed in the traditional job or gig-economy context (Kellogg et al., 2019). Flexibility, control, worker agency, resistance, fairness, and contextual variation are identified as recurring concerns in algorithmic management research (Chigbu, 2026). Studies also conceptualize algorithms as a contested terrain of organizational control rather than as neutral technological systems (Kellogg et al., 2019). Although artrepreneurs differ from conventional gig workers, both operate in environments where access to opportunities can increasingly depend on digital systems that they do not fully govern.

The literature therefore supports neither technological determinism nor an uncomplicated empowerment narrative. Rather, artrepreneurs appear to operate under constrained and negotiated agency. They retain capacities for interpretation, adaptation, experimentation, diversification, and resistance, but exercise these capacities within infrastructures characterized by externally defined rules, metrics, asymmetric information, and technical architectures.

The processes described above correspond to the platform-agency dimension of the framework. Platform agency concerns the capacity of artrepreneurs to interpret platform rules, metrics, affordances, and algorithmic conditions, experiment with platform practices, and adapt their strategies in response to observed outcomes. Algorithmic negotiation is therefore a central mechanism through which platform agency is exercised. It connects platform governance and algorithmic conditions to the broader configuration of creative, relational, and entrepreneurial agency.

## 7 Platform adaptation, heterogeneity and artistic autonomy

Algorithmic adaptation becomes particularly consequential when platform requirements begin to influence creative practices. Different platforms favor different content formats, posting frequencies, styles, and forms of audience interaction. Short-form video, personal storytelling, frequent posting, or visual consistency may become strategically valuable. Artists may therefore adapt not only how they promote their work but also how they package and, in some cases, produce it.

Platform conditions vary substantially across digital environments, and algorithmic dependence is therefore not uniform. Discovery and attention platforms may make recommendation systems, visibility metrics and content circulation particularly consequential. Streaming platforms and consumption devices may increasingly focus on algorithm-based recommendations, content finding and sustained users' attention. Marketplaces connect artists with buyers while imposing rules concerning search visibility, pricing, transactions and platform participation. Artist-controlled websites and other owned channels offer more control over the audience access and communication, although they provide less algorithmically generated discovery. These differences mean that the association between platform engagement and entrepreneurial agency can vary according to algorithmic dependence, income dynamics, audience portability constraints on creative production (Nieborg and Poell, 2018; Poell et al., 2021).

This creates a tension between platform participation and the preservation of artistic autonomy. Adaptation is not inherently problematic; entrepreneurship necessarily involves responding to changing environments. It becomes an issue when platform metrics become increasingly influential in shaping creative decisions. Optimisation for visibility can thus have an impact on art production without being the determining factor.

This is consistent with research on digital creative work. Research highlights how content creators negotiate professional identities and expectations among audiences, platforms and commercial partners (Hoose and Rosenbohm, 2023). Similarly, some new questions that have arrived around creative ownership, copyright, governance and the economic value of creative work in the generative AI context are being explored in recent studies (Whitaker, 2024).

These developments suggest that technological infrastructures increasingly affect not only the circulation of creative work but also questions of authorship, identity, ownership, and value. The real issue here, then, isn't so much if adaptation is necessary but how artrepreneurs can reconcile participation in platform economies with maintaining artistic autonomy and identity.

These dynamics relate to the creative-agency dimension of the framework in particular. Creative agency relates to the ability to retain artistic integrity and to make decisions about artistic practice, acknowledging that the platform formats, metrics and visibility demands may affect these decisions. Platform adaptation does not remove creative agency but establishes a context in which it is continually negotiated. Concurrently, the diversity of platforms can result in different levels of platform agency and creative agency in different infrastructural contexts.

## 8 Developing the algorithmic artrepreneur concept: boundaries and agency

Having established the conceptual framework as a synthesis of the reviewed literature, this section clarifies the conceptual boundaries of the algorithmic artrepreneur and explains the relationships among its four dimensions of agency.

Not every artist who uses social media is necessarily an algorithmic artrepreneur. An artist who maintains a social media account primarily for occasional information sharing or promotion would not necessarily fall within the concept. The concept starts to gain analytical value when it becomes frequent feature in the decision making process as far as creative presentation, audience development, networking, monetisation, opportunity seeking, career planning, etc. are concerned. Economic activity is also not enough since the arts entrepreneurship can also offer cultural, social, professional and symbolic value (Hausmann and Heinze, 2016; Martínez et al., 2026). The term therefore does not just mean artists that work with digital media, but artist entrepreneurs whose agency is produced in a meaningful way through continuous interaction with infrastructures of platforms.

The four dimensions in the framework are analytically separable but inter-constitutive. Creative agency is the ability to develop and make decisions about artistic practice. Platform agency is the ability to comprehend, interpret, navigate, try out, and react to platform-specific rules, metrics, affordances, and algorithmic conditions. Relational agency is about audience building and community, network and collaboration development and maintenance. Entrepreneurial agency is the ability to convert creative and relational resources into economic, professional, cultural and social value.

These dimensions are not to be seen as separate skills, which are performed in a linear order. Platform agency can impact creative agency when the norms, formats and metrics of the platform impact the creation or presentation of artistic work. It can also provide relational agency to artists in the process of identifying, connecting and engaging audiences and networks. Relational agency can be a part of entrepreneurial agency when audiences, communities and collaborations are translated into professional, cultural, social and/or economic value. Entrepreneurial goals can influence platform selection, audience-building strategies, and creative approaches, which can in turn affect the platform's selection. The four dimensions are thus not arranged in a hierarchy but in a circular and recursive arrangement in the framework.

### 8.1 Practical implications

The four-dimensional framework also defines the competencies related to each dimension of today's art entrepreneurship. Platform agency demands platform literacy, analytics and metrics interpretation, experimentation with platform affordances and platform governance knowledge. Relational agency is an activity of audience development, community building, networking and collaboration. Entrepreneurial agency is intellectual-property sensitivity, diversification of income, monetization, and the capacity to convert creative and relational resources into various kinds of value. It is important to reflect critically on the potential for platform optimization to strengthen or limit artistic identity and creative autonomy in creative agency. In all of these areas, platform diversification and direct audience relationships can help alleviate reliance on any single platform infrastructure. The competencies can be applicable to arts entrepreneurship education, professional-development programmes, and practitioner training, as they focus on the skills needed to work and navigate in platformized creative settings, rather than just using digital platforms.

## 9 Current gaps and future developments

The synthesis identifies four major areas requiring further research.

### 9.1 Algorithmic agency in arts entrepreneurship

Algorithmic management and creator labor research is growing but there is little development of the specific implications for arts entrepreneurship. Further investigations should explore how artists are learning about algorithms, developing strategies for working with them, and altering, diversifying, or resisting them.

Evaluating algorithmic dependence in comparative studies of music, visual arts, performing arts, crafts, design and other artistic areas could help identify if there is a difference in algorithmic dependence between disciplines and platforms. Comparative research should also examine how platform type and artistic discipline condition the relative importance of creative, platform, relational and entrepreneurial agency.

### 9.2 Visibility labor and artistic autonomy

Visibility labor remains insufficiently conceptualized as a distinct component of artrepreneurial work. How much time and mental effort do artists devote to the digital visibility? Does optimizing the platform decrease time for creative production? When does strategic adaptation become seen as creative compromise?

The following questions can link in research on creative labor with research on platform work. Future studies should also examine whether visibility labor produces durable audience relationships and entrepreneurial value, rather than assuming that greater visibility necessarily produces greater career success.

### 9.3 Unequal capacities for algorithmic navigation

Artists do not have equal resources for navigating digital platforms. The capacity to handle the uncertainties of algorithms might be influenced by digital literacy, financial capital, equipment, alternative income sources, and managerial support.

There is therefore a need for further research to investigate if platformization brings democracy to the practices of artistic entrepreneurship or reproduces existing inequalities in new and different ways of digital advantage. Such inequalities may affect not only access to platforms but also the capacity to interpret algorithms, experiment with platform strategies and convert visibility and relationships into entrepreneurial value.

### 9.4 Platform diversification and sustainable careers

Artrepreneurs who solely rely on one platform are at risk of shifts in algorithms, policies, monetisation models and audience access. Future research should examine how platform diversification, direct relationships with audiences, websites, memberships, licensing, commissions, offline networks, and multiple revenue streams can strengthen entrepreneurial resilience.

Longitudinal research is therefore needed to examine when visibility and engagement are converted into durable audience relationships, economic and professional value, artistic satisfaction and sustainable creative careers.

## 10 Discussion

The literature reviewed here reveals a central paradox in contemporary artrepreneurship. Digital platforms could also increase the possibilities for audience access, market engagement, collaboration, and revenue generation—and introduce new forms of infrastructural dependency for the production of creative entrepreneurship. Platformization thus calls into question the notion of increased entrepreneurial independence with increased digital access.

The framework captures this tension by placing platform governance and algorithmic conditions as the setting in which various modes of entrepreneurial agency are deployed. The literature reviewed in the previous sections can be mapped to the various elements of this framework: arts entrepreneurship as a foundation for creative and entrepreneurial agency; platformization as an infrastructural condition in which these forms of agency are enacted; visibility research as a focus on the relationship between platform exposure, relational processes, and value creation; and research on algorithmic governance as a focus on negotiation and adaptation processes through which platform agency is enacted. Platform heterogeneity also suggests that these relationships are not necessarily the same in all digital environments.

The evidence that is emerging also indicates that algorithmic governance is not about removing artists' or entrepreneurs' agency. Creators continue to interpret, adapt, experiment, diversify, and resist. These capacities are, however, performed in contexts where recommendation systems are opaque, metrics are used to measure, norms specific to platforms and resources are unevenly distributed. Therefore a sociologically useful interpretation is one that is characterized by constrained and negotiated agency rather than technological determinism or a straightforward digital empowerment.

The algorithmic artrepreneur is conceptually valuable insofar as it moves the conversation from whether artists use digital platforms to what entrepreneurial agency is becoming. The four dimensional framework suggests that agency, both creative and platform, relational and entrepreneurial are not separate and distinct capabilities but are mutually constitutive. Platform conditions can impact creative decisions, platform agency can shape relational opportunities, relational resources can be translated into entrepreneurial agency into many forms of value and entrepreneurial objectives can then reshape platform and creative strategies. Algorithmic negotiation is thus an iterative process of interpretation, experimentation, learning, adaptation and resistance, if needed. This view brings arts entrepreneurship scholarship to a new level by recognizing the platform less as an instrument of entrepreneurial action, and more as an infrastructure that structures the conditions for entrepreneurial action to come into being, become visible and have impact. The visibility generation is thus only the first component of sustainable artrepreneurship: in addition, it is necessary to be able to deal with platform dependence without losing the sense of professional identity, artistic autonomy and meaningful control of the creative production.

## 11 Conclusion

The platformization of creative work has changed the context within which contemporary artrepreneurs create, communicate and search for economic opportunities. Digital platforms can increase audience access, networking, audience visibility and visibility labor, generate infrastructural dependence and metric pressure, and also create algorithmic uncertainty. The conceptual framework developed in this review brings these conditions together by linking platform governance and algorithmic negotiation to four interrelated dimensions of artrepreneurial agency and to subsequent processes of audience development, value creation, and career sustainability.

This Mini Review introduces the notion of the algorithmic artrepreneur to capture this changing condition. The algorithmic artrepreneur is neither simply an artist who uses digital platforms nor an entrepreneur controlled by algorithms. Instead, it refers to an artist-entrepreneur who continuously exercises and negotiates creative, platform, relational and entrepreneurial agency within platform infrastructures.

Three tensions are particularly noteworthy:

(1) the tension between visibility as entrepreneurial opportunity and as labor;
(2) the tension between adaptation to platforms as an entrepreneurial strategy and as a creative constraint; and
(3) the tension between digital access as a democratization and as an infrastructural dependence. The focus of future studies should therefore move from the question of whether artists engage in social media, to how platform governance changes the work of the artist, entrepreneurial agency, inequality and career sustainability.

The central challenge of contemporary artrepreneurship is therefore no longer simply gaining access to digital platforms, but sustaining creative and entrepreneurial autonomy within infrastructures that increasingly organize visibility, audience access, value and opportunity. The contribution of the algorithmic artrepreneur concept lies not in naming artists who use digital platforms, but in providing a framework for understanding how entrepreneurial agency is reorganized when creative production, visibility, audience relationships and value creation become increasingly mediated by platform infrastructures.
